# Comparison of Three Different Internal Brace Augmentation Techniques for Scapholunate Dissociation: A Cadaveric Biomechanical Study

**DOI:** 10.3390/jcm10071482

**Published:** 2021-04-02

**Authors:** Il-Jung Park, Dohyung Lim, Mauro Maniglio, Steven S. Shin, Seungbum Chae, Victor Truong, Michelle H. McGarry, Thay Q. Lee

**Affiliations:** 1 Orthopaedic Biomechanics Laboratory, Congress Medical Foundation, Pasadena, CA 91105, USA; dli349@sejong.ac.kr (D.L.); maniglio.mauro@gmail.com (M.M.); sbchae@cu.ac.kr (S.C.); victor_trng@yahoo.com (V.T.); michelle@congressmedicalfoundation.org (M.H.M.); tqlee@congressmedicalfoundation.org (T.Q.L.); 2 Department of Orthopaedic Surgery, College of Medicine, The Catholic University of Korea, Seoul 06591, Korea; 3 Department of Mechanical Engineering, Sejong University, Seoul 05006, Korea; 4 Department of Orthopaedics and Traumatology, Inselspital Bern, University Hospital, 3010 Bern, Switzerland; 5 Department of Orthopaedics, Cedars-Sinai Health System, Los Angeles, CA 90045, USA; steven.shin@cshs.org; 6 Department of Orthopaedic Surgery, Daegu Catholic University Medical Center, Daegu Catholic University School of Medicine, Deagu 42472, Korea

**Keywords:** biomechanical characteristics, internal bracing, scapholunate interosseous ligament

## Abstract

Internal bracing (IB) is an augmentation method using high-strength nonabsorbable tape. However, there is no detailed information about the direction, location, or number of IBs required for scapholunate interosseous ligament (SLIL) injury repair. Thus, this study compared the biomechanical characteristics of short-transverse IB, long-oblique IB, and the combination of short-transverse and long-oblique (Combo) IB for SLIL injury in a biomechanical cadaveric model. We prepared nine fresh-frozen full upper extremity cadaveric specimens for this study. The scapholunate distance, scapholunate angle, and radioscaphoid angle were measured using the MicroScribe digitizing system with the SLIL intact, after scapholunate dissociation and the three different reconstructions. Three-dimensional digital records were obtained in six wrist positions in each experimental condition. Short-transverse IB had a similar effect compared with long-oblique IB in addressing the widening of the scapholunate distance. However, both were less effective than Combo IB. For scaphoid flexion deformity, short-transverse IB had minimal effect, while long-oblique IB had a similar effect compared to Combo IB. Combo IB was the most effective for improving distraction intensity and rotational strength. This study provides important information about the biomechanical characteristics of three different IB methods for SLIL injury and may be useful to clinicians in treating scapholunate dissociation.

## 1. Introduction

Surgery for scapholunate interosseous ligament (SLIL) injuries is determined based on the time from injury, extent of carpal instability, and presence of secondary changes in the carpus. The optimal surgical treatment for chronic scapholunate instability with an irreparable SLIL (but without osteoarthritis) is yet to be determined [1]. Many reconstructive procedures have been described in the literature. Some of the techniques currently used include dorsal capsulodesis, bone-ligament-bone autografts, reduction association with a screw of the scapholunate joint (RASL), the scapholunate axis method (SLAM), and a variety of tendon reconstruction methods [2,3,4,5,6,7,8]. However, the outcomes of these techniques are unpredictable, and there is no single most effective surgical procedure for chronic scapholunate instability.

Internal bracing (IB) is an augmentation method using high-strength nonabsorbable tape, and it provides immediate enhanced strength and support during the critical time of ligament healing [9,10]. IB augmentation has recently been applied in orthopedics; however, it has not been widely applied in the field of hand surgery. Therefore, there is a paucity of studies in the field of biomechanics investigating the characteristics of IB for SLIL injury [11]. Recently, a study reported that SLIL repair with IB augmentation demonstrated significantly higher strength than SLIL repair without augmentation [12]. However, the study did not provide detailed information about the direction, location, and number of IBs.

Scapholunate dissociation refers to an abnormal orientation of the scaphoid relative to the lunate and implies severe injury to the SLIL and other secondary stabilizing ligaments. The goal of the reconstruction is to limit the widening of the scapholunate distance and flexion deformity of the scaphoid to prevent future scapholunate advanced collapse arthritis. We performed a biomechanical comparison of short-transverse IB, long-oblique IB, and the combination of short-transverse and long-oblique (Combo) IB in a controlled laboratory cadaveric model. We established the three following hypotheses. First, short-transverse IB is more effective than long-oblique IB for addressing the widening of the scapholunate distance. Second, long-oblique IB is more effective than short-transverse IB for addressing scaphoid flexion deformity. Finally, the Combo IB is the most effective in treating scapholunate dissociation. This study aimed to investigate the effects of three different IB methods on addressing the widening of the scapholunate distance and scaphoid flexion deformity for scapholunate dissociation in a cadaver model.

## 2. Materials and Methods

We prepared 9 fresh-frozen full upper extremity cadaveric specimens (6 males and 3 females; mean age 63 (range, 58–69) years) for this study. All specimens were intact macroscopically and exhibited no gross pathological abnormalities. Each specimen was thawed at room temperature for 12 h before preparation. All surgical procedures were performed by a single senior surgeon to minimize variation in technique.

### 2.1. Specimen Preparation

Midline dorsal and volar forearm incisions were made, and skin flaps were elevated. All soft tissues proximal to the hand were dissected from the specimen, except for the wrist capsule, interosseous membrane, and tendons. The remaining tendons were the wrist flexors (flexor carpi radialis (FCR), flexor carpi ulnaris (FCU)), wrist extensors (extensor carpi radialis brevis (ECRB), extensor carpi radialis longus (ECRL), extensor carpi ulnaris (ECU)), and the deep flexors of the fingers (flexor digitorum profundus (FDP; four tendons)). The specimens were kept moist with normal saline throughout preparation and testing. The forearm was fixed in neutral rotation (with the humerus positioned vertically and the elbow at 90°) with a K-wire. The forearm was subsequently transected 16 cm from the tip of the radial styloid and potted in a 2-inch polyvinylchloride pipe measuring 6 cm in length using plaster of Paris. Care was taken to ensure neutral alignment in both the coronal and sagittal planes. Running, locking Krackow stitches were placed in each individual tendon using a 2-0 braided suture to allow subsequent loading. The ECRB and ECRL tendons were sutured together, and the four FDP tendons were sutured side to side to load them equally.

**Figure 1:**
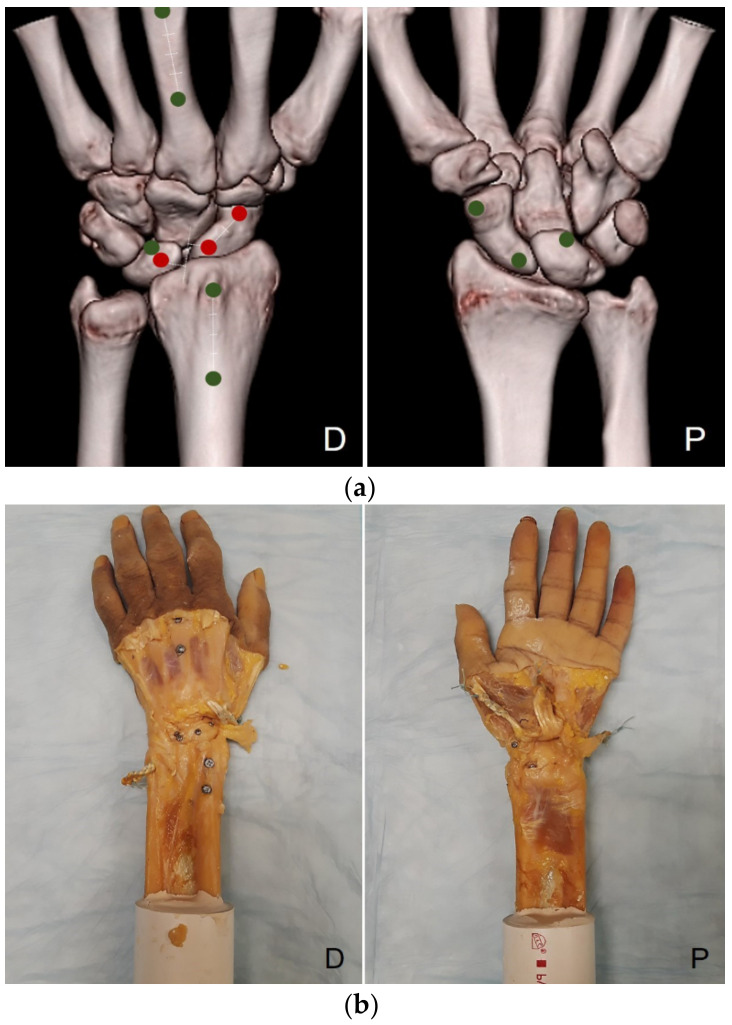
A consistent protocol utilizing anatomical landmarks was employed to place 11 digitizing markers. The markers on a computed tomography scan (**a**) and the cadaver (**b**). D, dorsal aspect; P, palmar aspect.

A consistent protocol utilizing anatomical landmarks was employed to place 11 digitizing markers on each specimen. The extensor retinaculum was opened between the third and fourth compartments, and a dorsal capsulotomy was performed. Four markers were made on the scaphoid, three on the lunate, two on the distal radius, and two on the third metacarpal bone (Figure 1). In the scaphoid, two markers were made on the dorsal and palmar sides, respectively. The dorsal proximal marker was located 5 mm from the scapholunate joint line in the radioulnar direction and the center of the scaphoid in the proximodistal direction. The dorsal distal marker was located 10 mm from the dorsal proximal marker along the long axis of the scaphoid. The palmar proximal and distal markers were located at the scaphoid proximal pole and the scaphoid tuberosity, respectively. These markers were the two palmar convexities of the scaphoid in the sagittal plane. In the lunate, two markers were made on the dorsal side and one on the palmar side. The dorsal proximal marker was located 5 mm from the scapholunate joint line and the center of the lunate. The dorsal distal and palmar markers were located on the most distal ulna side of the lunate. The line connecting these two markers was parallel to a line joining the two distal horns of the lunate in the sagittal plane. Two markers on the scaphoid and one marker on the lunate were designated as the holes for the IB reconstructions. The authors used calipers for marker placement and endeavored to ensure reproducibility.

### 2.2. Biomechanical Testing

**Figure 2:**
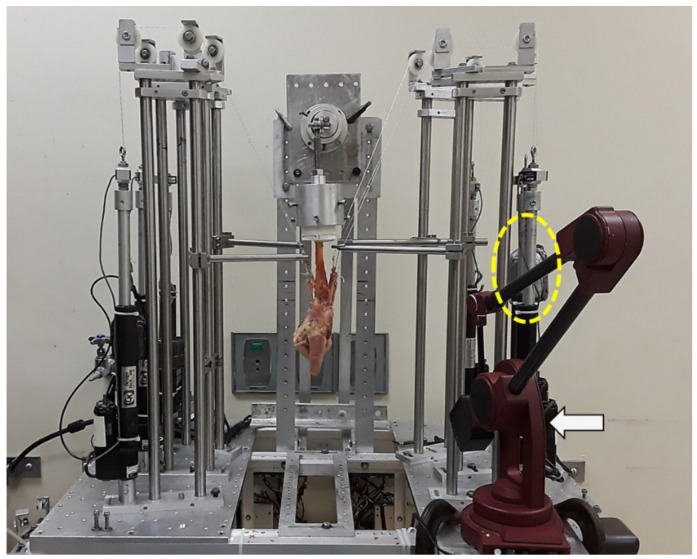
Specimens were tested using a custom wrist testing system designed to obtain carpal kinematic measurements. With the weights applied (yellow dotted circle), each wrist reached the maximum excursion that could be achieved in the desired wrist direction. The kinematic measurements were taken by digitizing the marker positions using a MicroScribe 3D digitizer (white arrow).

We used a model similar to those used in previous studies [1,13,14,15]. Specimens were tested with a custom wrist testing system designed to obtain carpal kinematic measurements under custom loading protocols (Figure 2). The potted forearm was securely mounted into a custom fixture that simulated 90° of elbow flexion.

For muscle loading, we prepared to suspend weights to simulate different wrist positions. The lines attached to the Krackow stitches of each individual tendon were positioned through guide rods to achieve physiologic lines of pull. Small metal hooks were used to suspend weights from the prepared tendons. Wrist flexion was created with 10-N weights applied to both the FCR and FCU tendons, and extension was created with a weight of 10 N on the ECU and 10 N on the combined ECRL/ECRB tendons. Ulnar deviation (UD) was created with 10 N on both the FCU and ECU tendons. Radial deviation (RD) was created with 10 N on the FCR tendons and 10 N on the combined ECRL/ECRB tendons. A clenched fist was simulated by applying a 20-N weight on the four FDP tendons after wrist extension (10 N on the ECU and 10 N on the combined ECRL/ECRB). With the loads applied, each wrist reached the maximum excursion that could be achieved in the desired wrist direction. The magnitude of these loads does not exactly match those used by Pollock et al. [1], as we observed that different amounts of weights were required to create the appropriate wrist motion. This study was designed with a load-controlled test. The load was the same for all specimens; however, the degree of motion achieved varied slightly between specimens, depending on the cadaver wrist and soft tissues.

Subsequently, the wrist flexors/extensors were fully loaded, and kinematic measurements were taken by digitizing the marker positions using a MicroScribe three-dimensional (3D) digitizer (Revware Inc., Raleigh, NC, USA; accuracy, 0.3 mm). It is a precision contact-based 3D digitizing device, which can be used to measure and capture 3D data points from physical objects. Before the measurements, we confirmed that the scapholunate distance, scapholunate angle, and radioscaphoid angle at the neutral position were within the normal range. If the measured value was not within the normal range, the specimen and markers were rechecked. The scapholunate distance, scapholunate angle, and radioscaphoid angle were obtained with the SLIL intact wrist in the neutral, flexion, extension, UD, RD, and clenched fist positions, sequentially. Each measurement was performed twice, and the data were averaged. Repeatability was checked; a third trial was performed if the difference between the first two trials was >1 mm or >1°.

The SLIL is the primary stabilizer, and the volar radioscaphocapitate ligament is a secondary stabilizer of the scapholunate articulation [16]. We sharply dissected off all the three (dorsal, membranous, and palmar) portions of the SLIL using a scalpel followed by the division of the volar radioscaphocapitate ligament to establish the condition of scapholunate dissociation. After sectioning both the SLIL and radioscaphocapitate ligaments, the scapholunate complex was grossly unstable. Thereafter, the scapholunate distance, scapholunate angle, and radioscaphoid angle were measured in six wrist positions in the same manner as for the SLIL intact wrist.

**Figure 3:**
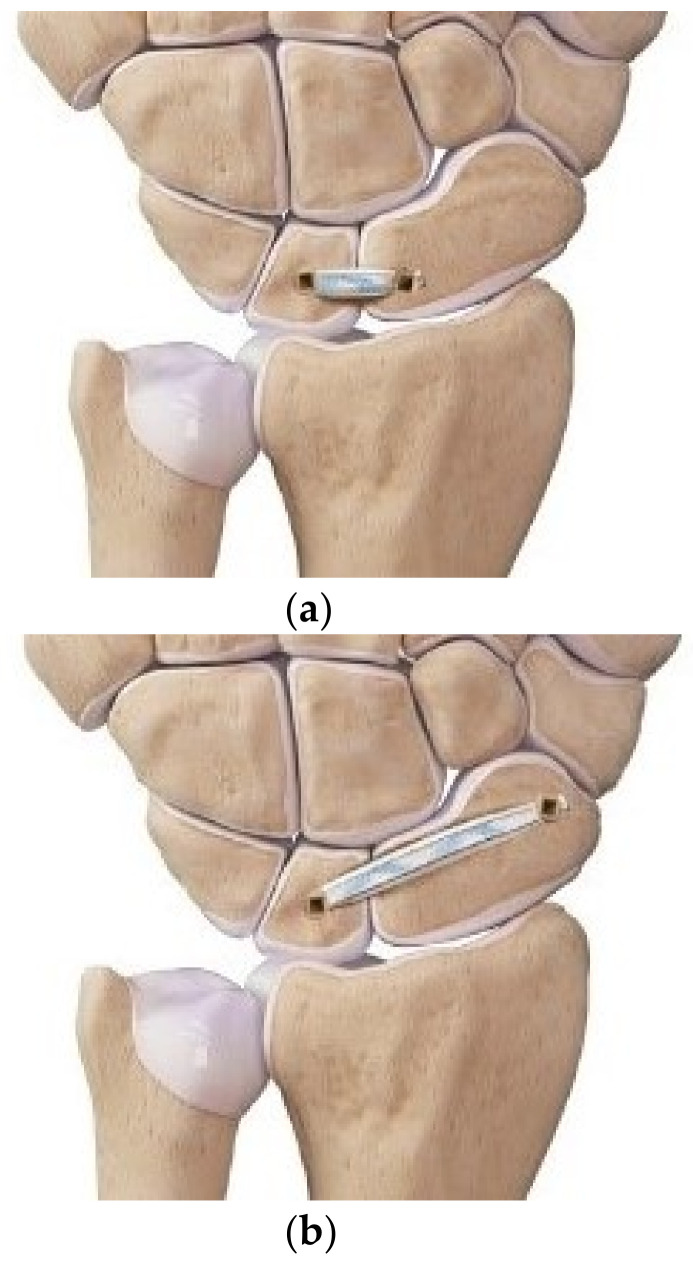

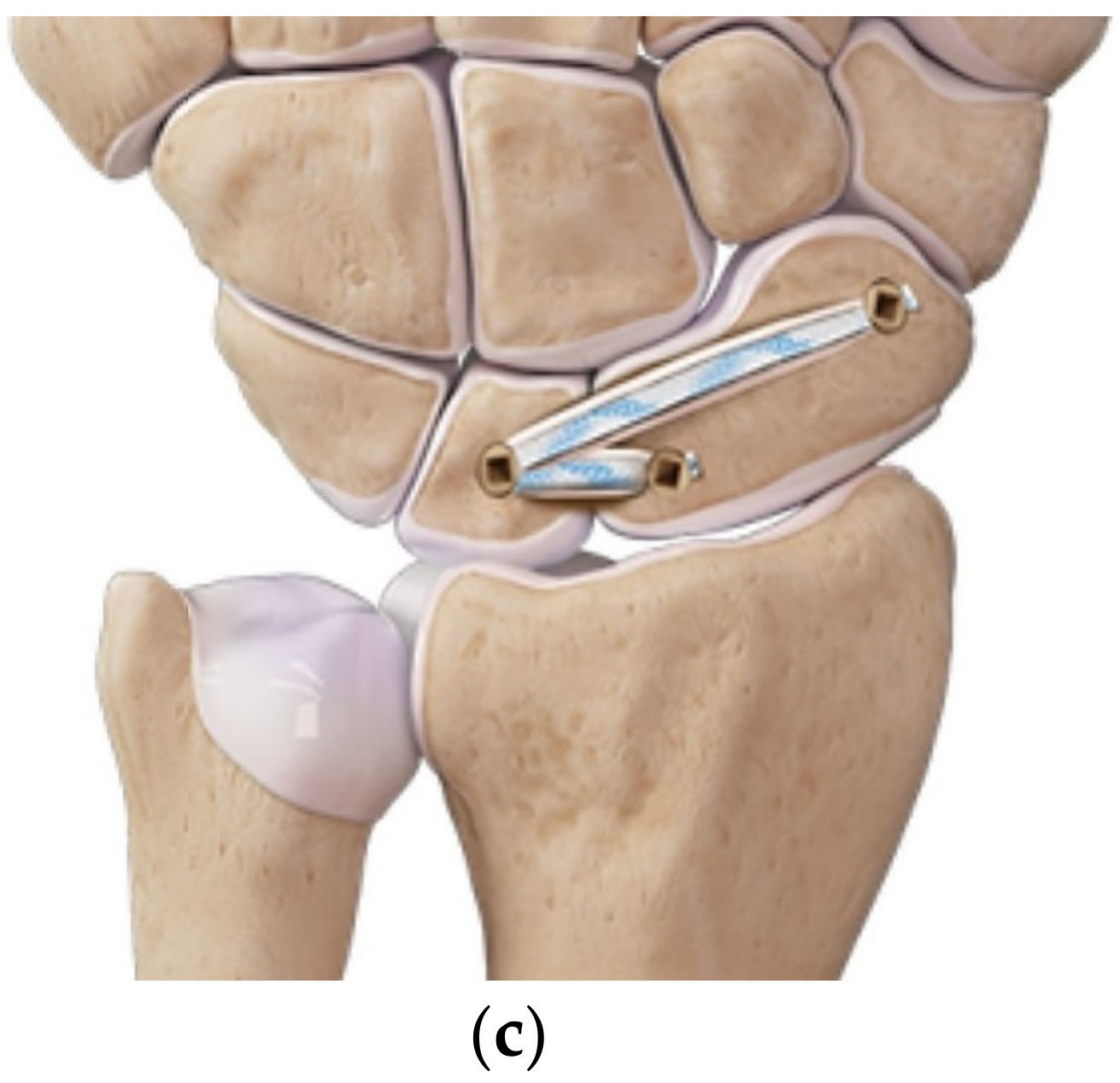
Schematic diagrams of the three different reconstructions. Short-transverse internal bracing (IB) (**a**), long-oblique IB (**b**), and Combo IB (**c**). This image was provided courtesy of Arthrex, Naples, Florida, 2021.

**Figure 4:**
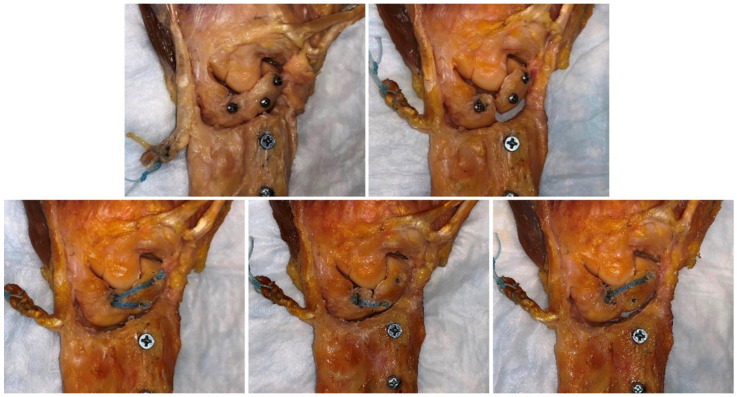
There were five testing states in this experiment: with the SLIL intact, after scapholunate dissociation (all SLIL and the radioscaphocapitate ligament cut off), and after the three different reconstructions. Regarding the order of reconstruction, Combo IB was performed first, and subsequently, long-oblique IB and short-transverse IB were randomly selected. SLIL, scapholunate interosseous ligament.

After the measurement of the scapholunate dissociation wrist, three different reconstructions were performed (Figure 3). Drill holes for IB (FiberTape; Arthrex, Naples, FL, USA) were created using markers made during the preparation of the specimen. After removing the markers, drill holes were made using a 3.0 mm drill bit. IB reconstructions were performed using suture anchors (3.5-mm DX SwiveLock SL; Arthrex), and we tried to insert the anchors at 90 degrees as inserting at an obtuse angle could reduce the pullout strength. Regarding the order of reconstruction, Combo IB reconstruction was performed first, and subsequently, long-oblique and short-transverse IB reconstructions were randomly selected (Figure 4). The measurements were repeated under the same conditions as for the intact SLIL and scapholunate dissociation after reconstruction.

### 2.3. Statistical Analysis

Previously obtained pilot study data were used to perform a sample size calculation. To obtain an α of 0.05 and power of 0.8, a minimum sample size of 9 specimens was deemed adequate to demonstrate a statistically significant difference. The Wilcoxon signed-rank test was employed to compare the biomechanical characteristics of intact SLIL, scapholunate dissociation, short-transverse, long-oblique, and Combo IB. *p <* 0.05 was considered to indicate statistical significance.

## 3. Results

The average angle of each wrist position during testing was as follows: neutral (extension 3.1° ± 0.6°/RD 11.1° ± 2.1°), flexion (flexion 59.8° ± 2.4°/UD 32.9° ± 3.0°), extension (extension 54.5° ± 2.7°/RD 11.7° ± 2.0°), RD (extension 15.0° ± 2.5°/RD 11.6° ± 2.1°), UD (flexion 33.5° ± 3.0°/UD 41.9° ± 2.7°), and clenched fist (extension 28.9° ± 2.3°/RD 17.2° ± 2.7°). All parameters (scapholunate distance, scapholunate angle, and radioscaphoid angle) worsened after scapholunate dissociation and improved after the three different reconstructions in almost all wrist positions.

### 3.1. Scapholunate Distance

**Figure 5:**
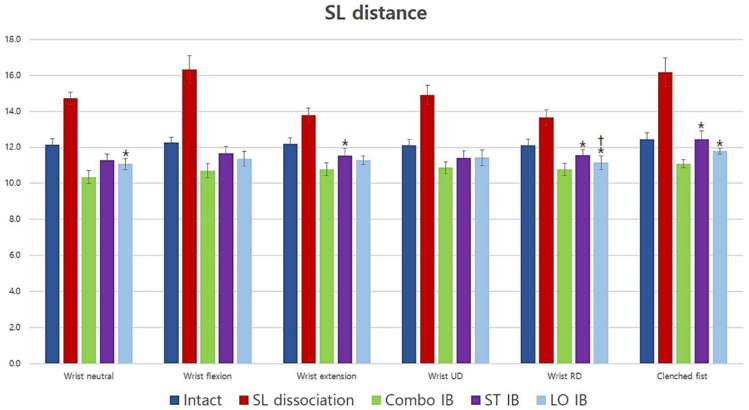
Average scapholunate distance for all conditions and positions (mm). Short-transverse and long-oblique IB had similar effects; however, both were less effective than Combo IB. *: Statistically significant difference compared to Combo IB (*p <* 0.05), †: Statistically significant difference compared with short-transverse IB (*p <* 0.05). SL: scapholunate; UD: ulnar deviation; RD: radial deviation; Combo: combination; IB: internal bracing; ST: short-transverse; LO: long-oblique.

The scapholunate distance significantly increased after scapholunate dissociation and improved after the three different reconstructions in all wrist positions. No statistically significant difference was observed between short-transverse and long-oblique IB except for RD (*p* = 0.038). The scapholunate distance of short-transverse IB was significantly wider than that of Combo IB for extension, RD, and clenched fist (*p* = 0.049, 0.012, and 0.011, respectively), and there was a similar but non-statistically significant pattern for neutral and flexion (*p* = 0.05 and 0.058, respectively). The scapholunate distance of long-oblique IB was significantly wider than that of Combo IB for neutral, RD, and clenched fist (*p* = 0.028, 0.042, and 0.028, respectively), and there was a similar but non-statistically significant pattern for flexion and UD (*p* = 0.05 and 0.058, respectively). In summary, no difference was observed between short-transverse IB and long-oblique IB for the scapholunate distance; however, both had wider scapholunate distances than Combo IB (Figure 5).

### 3.2. Scapholunate Angle

**Figure 6:**
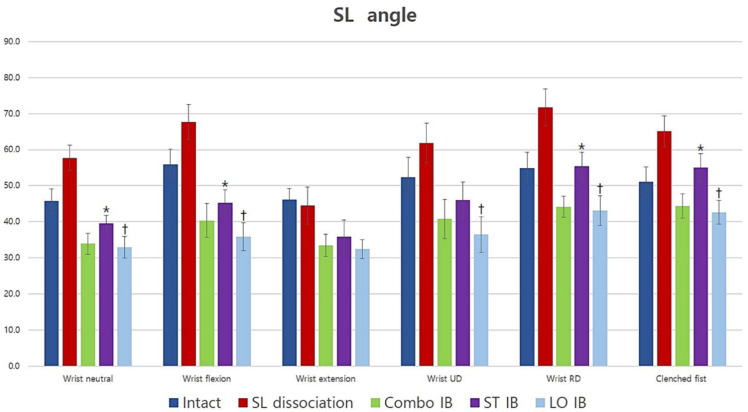
The average scapholunate angle for all conditions and positions (degrees). Short-transverse IB had less effect than long-oblique and Combo IB; however, long-oblique and Combo IB had similar effects. *: Statistically significant difference compared to Combo IB (*p <* 0.05), †: Statistically significant difference compared with short-transverse IB (*p <* 0.05) SL: scapholunate; UD: ulnar deviation; RD: radial deviation; Combo: combination; IB: internal bracing; ST: short-transverse; LO: long-oblique.

The scapholunate angle significantly increased after scapholunate dissociation in all positions except for extension (*p* = 0.515) and improved after the three different reconstructions in all wrist positions. Short-transverse IB demonstrated a significantly larger scapholunate angle than long-oblique IB for neutral, flexion, RD, UD, and clenched fist (*p* = 0.021, 0.008, 0.012, 0.008, and 0.008, respectively) except for extension (*p* = 0.214). Short-transverse IB demonstrated a significantly larger scapholunate angle than Combo IB for neutral, flexion, RD, and clenched fist (*p* = 0.021, 0.028, 0.008, and 0.008, respectively). No statistically significant difference was observed between long-oblique and Combo IB in all wrist positions. In summary, short-transverse IB had a larger scapholunate angle than long-oblique and Combo IB; however, no difference in scapholunate angle was observed between long-oblique and Combo IB (Figure 6).

### 3.3. Radioscaphoid Angle

**Figure 7:**
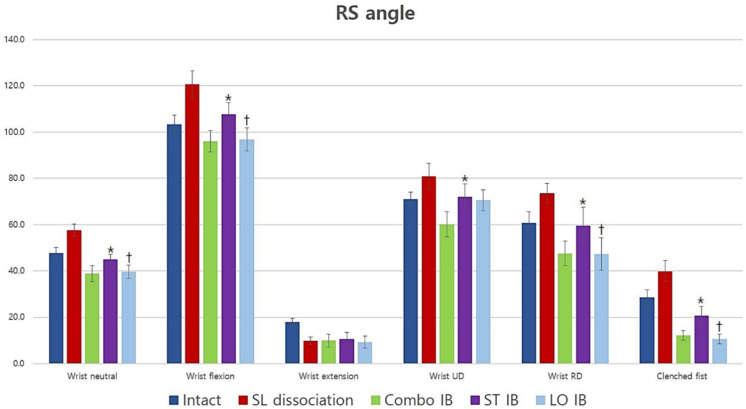
The average radioscaphoid angle for all conditions and positions (degrees). Short-transverse IB had less effect than long-oblique and Combo IB; however, long-oblique and Combo IB had similar effects. *: Statistically significant difference compared to Combo IB (*p <* 0.05), †: Statistically significant difference compared with short-transverse IB (*p <* 0.05). SL: scapholunate; UD: ulnar deviation; RD: radial deviation; Combo: combination; IB: internal bracing; ST: short-transverse; LO: long-oblique.

The radioscaphoid angle significantly increased after scapholunate dissociation in all positions except for clenched fist (*p* = 0.051) and improved after the three different reconstructions in all wrist positions except extension. The radioscaphoid angle of short-transverse IB was significantly larger than that of long-oblique IB for neutral, flexion, RD, and clenched fist (*p* = 0.028, 0.008, 0.021, and 0.008, respectively). The radioscaphoid angle of short-transverse IB was significantly larger than that of Combo IB for neutral, flexion, RD, UD, and clenched fist (*p* = 0.008, 0.008, 0.021, 0.038, and 0.038, respectively) except for extension (*p* = 0.262). No statistically significant difference was observed between long-oblique and Combo IB in all wrist positions. In summary, short-transverse IB had a larger radioscaphoid angle than long-oblique and Combo IB; however, no difference in radioscaphoid angle was observed between long-oblique and Combo IB (Figure 7).

## 4. Discussion

According to the study by Garcia-Elias et al., stage 3 and 4 SLIL injury comprised a complete, non-repairable ligament, with either a normally aligned scaphoid or reducible deformity [8]. In this setting, SLIL reconstruction may be considered, and several reconstructive procedures have been described. However, the outcomes of these reconstructions can be unpredictable, and there is no single most effective surgical procedure [14]. One reason many reconstructions have failed over the years is that the tendon graft stretches out and the fixation is weak [17,18,19,20]. Recently, reconstruction using the slip of the ECRB tendon with IB augmentation has been introduced; here, suture anchors with the combination of biologic tendon and synthetic suture tape are used to secure the central portion of the lunate to the dorsal proximal and distal poles of the scaphoid [21]. IB aims to reinforce biologic reconstruction with a suture to enable immediate biomechanical support and strength while the graft is incorporated [9,10,11]. It creates two limbs: a short-transverse limb that corrects the scapholunate distance and a long-oblique limb that corrects dorsal intercalated segment instability and scaphoid rotary subluxation. Both the scaphoid and lunate bony anchors are resistant and are solidly wedged into the tunnel with interference, leaving no weakness in the fixation points. Mullikin et al. [21] reported an excellent wrist range of motion and grip strength in a patient who, at the 2-year follow-up, was pain-free and had returned to all activities. Although there are a few clinical results for this technique, it is considered a method for overcoming the disadvantages of many other reconstructions.

There have been some biomechanical experiments of the wrist using radiographic evaluation [1,14,15,22,23,24,25]. These methods involve the use of radiographic images after creating the desired condition on the specimens. However, these methods require radiation shielding. True posteroanterior and lateral radiographs are needed, and if there is a rotational error in the radiographic position, it could affect the accuracy. In addition, there may be problems with inter- and intra-reliability when measuring parameters with the radiographs. The most important issue is that two-dimensional radiographs were used to measure a 3D problem. Carpal alignment can be best assessed in a 3D study [23]. This study used a MicroScribe 3D digitizer, which digitizes three points on the posts that are anchored into the radius, scaphoid, lunate, and third metacarpal bone. It records the location of the point it is touching whenever a foot pedal is depressed. Using these digitized points on each bone, custom coordinate systems can be created to make anatomic measurements for neutral, flexion/extension, RD/UD, and clenched fist. We used it with two measurements, and if the difference between the first two trials was >1 mm or >1°, a third trial was performed. Moreover, it is easy to use. The MicroScribe is one of the most reliable techniques for measuring the human skeleton [26].

Scapholunate instability is far more complex than a simple transverse diastasis between the scaphoid and lunate. The goal of the reconstruction is to limit the widening of the scapholunate distance and flexion deformity of the scaphoid to prevent future scapholunate advanced collapse arthritis. The following conclusions can be drawn from this study. Short-transverse IB has a similar effect to long-oblique IB in addressing the widening of the scapholunate distance. However, both are less effective than Combo IB. In addressing scaphoid flexion deformity, short-transverse IB has minimal effect, while long-oblique IB has a similar effect to that of Combo IB. Combo IB is the most effective for improving distraction intensity and rotational strength. The clenched fist position maximally stresses SLIL and has been shown to best evaluate scapholunate instability [1,27]. The results in the clenched fist position are as follows: regarding the scapholunate distance, no significant difference was observed between short-transverse and long-oblique IB; however, both had a wider scapholunate distance than Combo IB. Regarding the scapholunate angle and radioscaphoid angle, short-transverse IB had larger angles than long-oblique and Combo IB; however, no significant difference was observed between long-oblique and Combo IB. These results were exactly consistent with the result of the total experiment.

The SLIL comprises dorsal, membranous, and palmar regions. In a previous study, the palmar ligament had a yield strength of 120 N compared with the dorsal ligament, which exhibited a breaking strength of 300 N [28]. For this reason, the dorsal SLIL has traditionally been the primary focus of reconstruction techniques [29]. Recently, techniques that address both dorsal and volar ligaments have been introduced [23,30,31,32]. However, it remains unclear whether it is necessary to reconstruct the volar SLIL. Further research comparing dorsal-only reconstruction and combined dorsal and volar reconstruction is needed to determine the necessity of volar SLIL reconstruction.

Exclusive reconstruction of the dorsal ligament may lead to excessive tightening on the dorsal side and have the risk of hinge effect on the volar side [14,23]. In fact, the results of this study showed that the stability was stronger in all three IB reconstructions than the SLIL intact. However, we believe that early creep and delayed elongation could be expected as with all tendon-to-ligament reconstructions. Additionally, the stiffness caused by excessive tightening could be properly controlled by the operator. The most important pathogenesis of SLIL injury is the dorsal subluxation of proximal pole scaphoid with flexion deformity. Of course, the main goals of reconstruction are to limit the widening of the scapholunate distance and address the flexion of the scaphoid. However, among them, the authors believe that correction of the scaphoid flexion deformity is more important. Based on this, we consider that short-transverse IB is not suitable for the treatment of scapholunate dissociation. Additionally, we believe that Combo IB is the most effective for improving distraction intensity and rotational strength.

This study has some limitations. First, a time-zero cadaveric biomechanical study does not demonstrate in vivo clinical outcome over time. Thus, clinical postoperative strength and stiffness remain unknown. Moreover, we could not ascertain the long-term effects of IB in vivo; nevertheless, we are unaware of any published reports about humans’ adverse reaction to this synthetic material. Second, carpal bones have a complex relationship that depends on the wrist position and the direction of motion. The three parameters (scapholunate distance, scapholunate angle, and radioscaphoid angle) alone cannot comprehensively reflect the static and dynamic relationships of the carpal bones. However, no parameter better reflects the relationships of the carpal bone; therefore, most experiments regarding scapholunate dissociation use these parameters. Third, the scapholunate dissociation could be established following disruption in all secondary stabilizers. We reviewed many previous studies similar to our experiment. The method of establishing scapholunate dissociation differed slightly among authors. Many authors transected the SLIL and volar radioscaphocapitate ligaments [1,15,16,23]. Some authors transected the SLIL, volar radioscaphocapitate ligament, and the scaphotrapeziotrapezoid joint capsule [8,14], or the SLIL and dorsal intercarpal ligament [22]. We transected the SLIL and volar radioscaphocapitate ligament similar to other authors; however, we are unsure whether true scapholunate dissociation was created without disrupting all secondary stabilizers. Finally, this study was designed with a load-controlled test. The load was the same for all specimens; however, the degree of motion achieved varied slightly between specimens, depending on the cadaver wrist and soft tissues. In general, the soft tissue conditions of the elderly are weaker than those of the young. Therefore, we tried to avoid specimens of individuals of too old an age (mean age 63 (range, 58–69) years). Clinically, the reconstructions are more geared for younger patients with higher demanding activities; in the setting of cadaveric specimens of the elderly category, we would expect that the joint space narrowing would lead to decreased displacement with load testing. However, in biomechanical testing, the age range is reasonable, and these are the limitations of the scientific experiments.

Despite these limitations, this study provides important information about the biomechanical characteristics of three different IB methods for SLIL injury and may be useful for treating scapholunate dissociation. Future studies could consider a clinical series using the Combo IB in cases of dynamic and static scapholunate dissociation.

## Data Availability

The data presented in this study are available on request from the corresponding author.
